# The complete mitochondrial genome of the long-horned caddisfly *Triaenodes tardus* (Insecta: Trichoptera: Leptoceridae)

**DOI:** 10.1080/23802359.2017.1398619

**Published:** 2017-11-06

**Authors:** Melanie M. L. Lalonde, Jeffrey M. Marcus

**Affiliations:** Department of Biological Sciences, University of Manitoba, Winnipeg, MB, Canada

**Keywords:** Illumina sequencing, mitogenomics, Trichoptera, Leptoceridae, TTG initiation codon

## Abstract

The long-horned caddisfly *Triaenodes tardus* Milne, 1934 (Leptoceridae), is a widespread herbivorous North American caddisfly found in both lentic and lotic habitats. Whole genome Illumina sequencing allowed the assembly of a complete circular mitogenome of 14,963 bp from *T. tardus* consisting of 73.4% AT nucleotides, 22 tRNAs, 13 protein-coding genes, two rRNAs and a control region in the ancestral insect gene order. *Triaenodes tardus COX1* features an atypical TTG start codon as in some lepdioptera and prokaryotes. Phylogenetic reconstruction places *T. tardus* as sister to *Sericostoma personatum* (Sericostomatidae) within a monophyletic Order Trichoptera, which is consistent with previous phylogenetic hypotheses.

Most caddisfly larvae (Insecta: Trichoptera) are benthic aquatic detritivores. However, caddisflies in family Leptoceridae have morphological adaptations for swimming and species in leptocerid genus *Triaenodes* are herbivorous (Gall et al. 2011). *Triaenodes tardus* Milne, 1934 is a widespread North American species (NatureServe 2017) whose larvae are found in littoral vegetation in lotic and slowly flowing lentic habitats (Schwiebert 2007). Here we present the first complete mitogenome for family Leptoceridae from *T. tardus.*

On 14–15 August 2015, a USDA blacklight trap (Winter 2000) was deployed to collect night-flying insects at the Living Prairie Museum (GPS 49.889607 N, −97.270487 W), 12.9 hectares of relict prairie in Winnipeg, Manitoba, Canada (Living Prairie Mitogenomics Consortium 2017). Two adult *T. tardus* were trapped (specimens: 2015.08.14.063, 2015.08.14.077; determined by morphology and *COX1* barcodes). Specimen 2015.08.14.077 was pinned and deposited in the Wallis Roughley Museum of Entomology, University of Manitoba (voucher JBWM0361497).

DNA was prepared (McCullagh and Marcus 2015) and sequenced by Illumina MiSeq (San Diego, CA) (Peters and Marcus 2017). The mitogenome of *T. tardus* (Genbank MG201852) was assembled by Geneious 10.1.2 from 8,257,770 paired 75 bp reads using an *Anabolia bimaculata* (Trichoptera: Limnephilidae) reference mitogenome (MF680449) (Peirson and Marcus 2017). Annotation was in reference to *A. bimaculata* and *Sericostoma personatum* (Trichoptera: Sericostomatidae, KP455290) mitogenomes (Dietz et al. 2015). The *T. tardus* nuclear rRNA repeat (Genbank MG201853) was also assembled and annotated using *A. bimaculata* (MF680448) (Peirson and Marcus 2017) and *Stenopsyche marmorata* (Trichoptera: Stenopsychidae, LC094265.1) reference sequences.

The *T. tardus* circular 14,963 bp mitogenome assembly was composed of 6,952 paired reads with nucleotide composition: 33.2% A, 13.8% C, 12.9% G, and 40.2% T. The gene composition and order in *T. tardus* is identical to all known trichopteran mitogenomes except for *Hydropsyche pellucidula* (Hydropsychidae) (Linard et al. 2017). *Triaenodes tardus COX1* features an atypical TTG start codon as in some Lepidoptera (Chen et al. 2012) and prokaryotes (Asano 2014). The mitogenome contains three protein-coding genes (*COX2, NAD4*, and *NAD5*) with single-nucleotide (T) stop codons, and two protein-coding genes (*ATP6* and *NAD3*) with two-nucleotide (TA) stop codons completed by post-transcriptional addition of 3′ A residues. All tRNAs have typical cloverleaf secondary structures except for trnS (AGN) where the dihydrouridine arm is replaced by a loop as determined in Mfold (Zuker 2003). The rRNAs and control region are typical for Trichoptera (Peirson and Marcus 2017).

We reconstructed a phylogeny using 13 mitochondrial protein coding genes from *T. tardus,* seven other trichopteran species, and species in related holometabolous insect orders. Each gene was aligned in CLUSTAL Omega (Sievers et al. 2011), concatenated, and analyzed by maximum likelihood (ML) and parsimony in PAUP* 4.0b8/4.0d78 (Swofford 2002) (Figure 1). ML phylogenetic analysis shows Trichoptera as monophyletic; places *T. tardus* as sister to *Sericostoma personatum*, consistent with previous phylogenetic hypotheses (Kjer et al. 2002); and the primitive lepidopteran *Micropterix calthella* (Micropterigidae) was found to be sister to the Trichoptera, while the Trichoptera + *Micropterix* clade was sister to the remaining Lepidoptera (Peirson and Marcus 2017). The trichopteran family Limnephilidae is not monophyletic in this analysis, which warrants further investigation.

**Figure 1.**
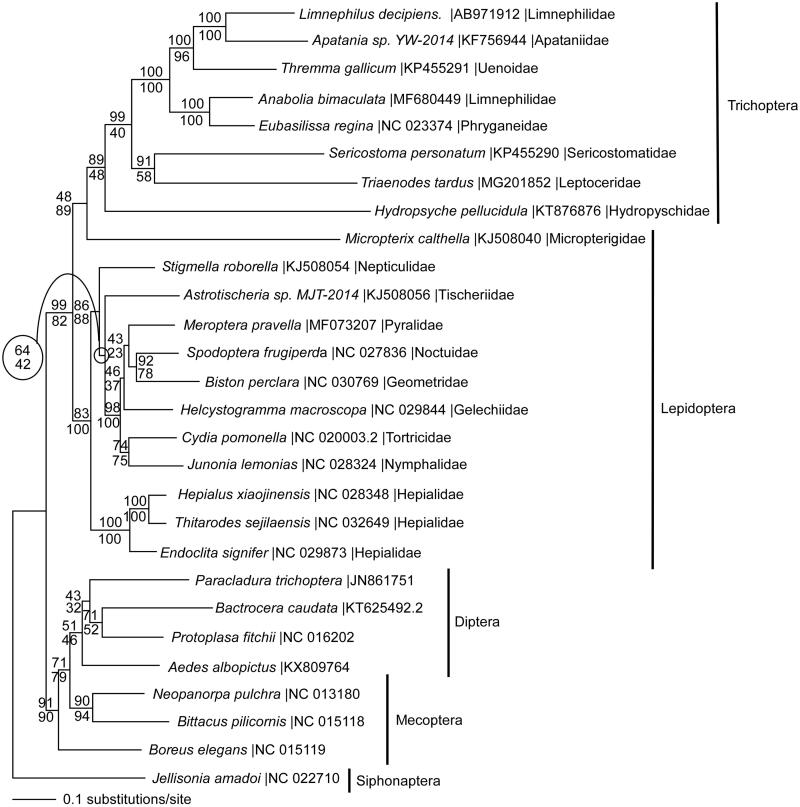
Maximum likelihood phylogeny (GTR + I + G model, I = 0.1960, G = 0.6200, likelihood score 175,277.12756) of *Triaenodes tardus* and other Trichoptera species with representatives from related insect orders Lepidoptera (moths and butterflies), Diptera (flies), Mecoptera (scorpionfiles), and Siphonaptera (fleas) based on 1 million random addition heuristic search replicates (with tree bisection and reconnection) of mitochondrial protein coding genes. One million maximum parsimony heuristic search replicates produced a single nearly identical tree (42,585 steps) except that *Micropterix* is the sister taxon to *Hydropsyche*, rather than to the entire trichopteran clade. Maximum likelihood bootstrap values are above nodes and maximum parsimony bootstrap values are below nodes (each from 1 million random fast addition search replicates).
